# Interactions between Polygenic Scores and Environments: Methodological and Conceptual Challenges

**DOI:** 10.15195/v7.a19

**Published:** 2020-09-21

**Authors:** Benjamin W. Domingue, Sam Trejo, Emma Armstrong-Carter, Elliot M. Tucker-Drob

**Affiliations:** a) Stanford University;; b) University of Wisconsin–Madison;; c) University of Texas at Austin

**Keywords:** polygenic score, gene–environment interaction

## Abstract

Interest in the study of gene–environment interaction has recently grown due to the sudden availability of molecular genetic data—in particular, polygenic scores—in many long-running longitudinal studies. Identifying and estimating statistical interactions comes with several analytic and inferential challenges; these challenges are heightened when used to integrate observational genomic and social science data. We articulate some of these key challenges, provide new perspectives on the study of gene–environment interactions, and end by offering some practical guidance for conducting research in this area. Given the sudden availability of well-powered polygenic scores, we anticipate a substantial increase in research testing for interaction between such scores and environments. The issues we discuss, if not properly addressed, may impact the enduring scientific value of gene–environment interaction studies.

Over the past decade, the world has witnessed a massive expansion of our ability to conduct biological inquiry into human behavior (Visscher et al. 2017). Genome-wide association studies (GWAS) (Pearson and Manolio 2008) have established that a broad array of behavioral traits (e.g., mental well-being, cognitive function, tobacco use, and risk-taking) and biomedical traits (e.g., height, body mass index, cholesterol, and cardiovascular disease) are highly polygenic (Boyle, Li, and Pritchard 2017; Chabris et al. 2015). Thus, population variation in these traits is attributable to many genetic variants, each individually exhibiting a relatively small effect. This has led many researchers to forego the study of specific genetic variants in favor of genome-wide composite measures (Dudbridge 2013). These composite measures, known as polygenic scores (PGSs), summarize the cumulative effects of many variants across the genome and aim to index an individual’s genetic liability for a given trait. PGSs constructed from large GWAS are robustly predictive of a sizable proportion of variance in consequential outcomes, such as educational attainment and lifespan (Cesarini and Visscher 2017; Lee et al. 2018; Sugrue and Desikan 2019).^1^ In fact, many PGSs are predictive of important biobehavioral and social science outcomes that were not the target of the original GWAS. Although PGS are neither pure (they may capture, e.g., correlated nongenetic factors [Morris et al. 2020]) nor universal (i.e., they may not generalize to environmental contexts not captured in the original GWAS from which they were constructed [Mostafavi et al. 2020]) measures, they have still sparked substantial interest. Many have argued that PGSs may advance our understanding of the behavioral and biomedical sciences (Belsky and Harden 2019; Conley and Fletcher 2017; Dudbridge 2016; Harden and Koellinger 2020). Sociologists, in particular, have begun to offer frameworks for thinking about how the discipline may benefit from such work (Freese 2018; Mills and Tropf 2020).

The increasing adoption of genetic approaches in social and behavioral science research has not diminished interest in the environment. Indeed, how social and environmental factors combine and interact with biological factors to produce individual differences is a question at the forefront of many research agendas in the social and behavioral sciences. Researchers have long posited that genetic effects likely vary as a function of environment (Feldman and Lewontin 1975).^2^ For example, in the twin study literature, there has been substantial interest in whether decompositions of observed variation in a phenotype into genetic and environmental components differ by socioeconomic context or age (Purcell 2002). Other research designs have tested interactions between measured genotypes (i.e., individual genetic variants) and environmental features. Although such an approach has intuitive appeal, it has proven technically challenging to implement (Duncan and Keller 2011). Some of the challenges of this research agenda may be attributable to unrealistically large expectations for effect sizes of individual variants and thus circumvented through use of PGSs. Yet, even when gene–environment interaction (GxE) results are robust and replicable, the interpretational and practical implications of such research can be unclear.

Polygenic scores are rapidly becoming widely available. Data sets such as the Health and Retirement Study (HRS; Ware et al. 2017), Add Health (Braudt and Harris 2018), and the Wisconsin Longitudinal Study (Okbay, Benjamin, and Visscher 2018) are posting preconstructed scores for use by researchers, and catalogs of polygenic scores are being made available (Lambert et al. 2020). This novel data resource may offer new and more robust avenues for exploration of GxE. However, challenges remain. Given the emergence of this new tool, we aim to provide timely guidance on how to conduct high-quality GxE research using PGSs. In this article, we have two main objectives. First, we outline several concerns associated with performing GxE research that future work may benefit from considering. Second, we offer some guidelines for designing, implementing, and interpreting high-quality GxE research using PGSs.

## The Standard GxE Model

We consider some outcome, *ϒ*, to be a function of an individual’s genotype, *G*, and some (potentially continuously varying) environmental exposure, *E*. We generically describe this data-generating model as

(1) E(ϒ∣…)=f(G,E).

Equation (1) accommodates both complex interplay between genotype and environment as well as outcomes that are not normally distributed (e.g., *ϒ* may have a Bernoulli distribution). We supplement this simple model with a few crucial assumptions. We assume that we have reasonable proxies available for *G* and *E* and some identifiable approximation to *f* (). We comment on each of these assumptions below.

With respect to *G*, we assume that we can characterize genetic influence on the trait as a PGS,

$${\text{PGS}}_{i}=\sum \beta_{j}{\left(N\text{ Alleles }\right)}_{ij},$$

that is, a sum wherein the number (*N*) of alleles (0, 1, or 2) that an individual *i* has for each single nucleotide polymorphism (SNP) *j* is weighted by the effect, *β*_*j*_, identified via GWAS. We note a few assumptions implicit in the above. We are focusing on traits that have a genetic architecture appropriately characterized by effects that are *additive with respect to one another* (although they may be nonadditive in terms of their potential to interact with environmental contexts) and dispersed over many loci. We view the assumption of additivity as an acceptable simplification given both the success of additive GWAS and the relative lack of strong empirical support for dominance or epistasis (i.e., gene–gene interaction) models (Polderman et al. 2015).

The assumption that genetic effects spread over many loci is not especially restrictive. Empirical work has indicated that many traits of interest in population health—body mass index, cardiovascular disease—are highly polygenic. Not all traits necessarily have this characteristic; consider, for example, monogenic diseases such as cystic fibrosis. However, as the sample size of the GWAS used to generate a PGS increases, weights (i.e., *β*_*j*_) for SNPs that are not relevant to the phenotype of interest will go toward zero; thus, a summative approach can still potentially be used in such cases. Moreover, much of our discussion still applies when using genetic predictors constructed from a smaller number of variants or even using a single variant allele count (e.g., Boardman et al. 2012; Rosenquist et al. 2015).

We also note that GWAS results (i.e., *β*_*j*_) are themselves potentially a function of both trait-specific biology and contextual features of the data used to derive them: for example, the social and policy landscape governing behavior of participants in the GWAS, selection issues associated with being a part of GWAS sample, et cetera. (Mostafavi et al. 2020; Pirastu et al. 2020). PGSs index the genetic propensity within the environmental context and demographic characteristics of participants in the original discovery GWAS on which the PGS is constructed.^3^ An interaction between PGS and environment may then indicate that the influence of genetic factors on the outcome is larger in some environments than others, that the sample in one environment is more similar to the sample from the discovery GWAS than in others, or some mixture of the two. This ambiguity regarding interpretation is important to keep in mind when findings from polygenic score research are interpreted. However, we focus the current article on inferential and statistical issues pertaining to the samples in which the PGSs are constructed and analyzed (i.e., we do not focus on the potential mismatch between that sample and the GWAS discovery sample).

With respect to *E*, we assume that researchers use specific measures of the environment, which we denote ENV. At present, research typically focuses on variation in measured environments that have relatively large main effects on *ϒ*. We consider this topic in detail later. In general, we emphasize that there are numerous challenges associated with identification of the appropriate ENV in GxE research (Boardman, Daw, and Freese 2013). The identification of appropriate ENV measures merits additional scrutiny in future work. Following selection of a candidate ENV, more questions follow. Are we measuring the environmental characteristic at the appropriate level (e.g., household vs. neighborhood vs. community)? Are we measuring a salient exposure given the respondents’ ages? Can we measure the environmental exposure of interest with high fidelity? Are the exposures and contexts of interest correlated with other, unmeasured, environmental or genetic variables that are themselves the driving forces in the identified GxE?

Finally, we assume that the unknown function *f* () is well approximated by a relatively simple model. In particular, many GxE studies aim to shed light on Equation (1) using regression models of the form

(2) E(ϒ∣…)=b0+b1⋅PGS+b2⋅ENV+b3⋅PGS⋅ENV+covariates.

The aim is to have Equation (2) elucidate key properties of the (unknown) data-generating process, even if Equation (2) is only a rough approximation of Equation (1). There are several concerns that apply to such regression models. We review two important issues that have been the subject of previous scrutiny below before then considering several novel issues of specific relevance when conducting GxE studies in the next section.

First, environmental exposures are typically partly endogenous (Jaffee and Price 2007), creating complex patterns of correlations between genes, focal environments, and other relevant exposures that lead to inferential challenges for the identification of GxE. We do not provide an in-depth treatment of this issue here as it has been discussed in depth elsewhere (Briley et al. 2019; Dudbridge and Fletcher 2014; Fletcher and Conley 2013). The question of endogeneity is, of course, closely related to the question of whether the measured environment that statistically moderates PGS effects has a causal effect. This is of course a crucial question; whether the effect is causal has direct implications for whether direct manipulation of that environment will produce changes in the genotype–phenotype association. Second, misspecification bias is a generic problem that introduces additional complexities in the case of interaction research. For example, care must be taken to distinguish between models containing interactions between two variables versus those with no interactions but nonlinear (e.g., quadratic) terms in one or both of the two variables (Lubinski and Humphreys 1990; MacCallum and Mar 1995). In particular, GxE research must also attend to the issue raised by Keller (2014) focusing on the covariates included in Equation (2). When covariates are included in Equation (2), specification error may result if additional interaction terms between the covariates and both *E* and the PGS are not included. This is because the main effects of the covariates are insufficient controls in the case where there is covariation between both the covariate and genotype or the covariate and the environment. Fortunately, there is a straightforward solution. Researchers simply need include the full suite of interaction terms between the PGSs and the covariates when estimating Equation (2).

## Study Design Issues in GxE Research

### The Environmental Exposure

#### The problem.

A great deal of research in the social sciences focuses exclusively on the effects of environments. For example, there is substantial interest in the effects of poverty, reflected primarily in the home environment of a young child, on the developing brain and related cognitive functioning (Duncan and Magnuson 2012; Johnson, Riis, and Noble 2016). GxE research has tended to emphasize environmental variables, like poverty, for which large main effects have been well documented (Barr et al. 2018; Gould et al. 2018; Musci et al. 2019). However, the environmental features having large main effects need not also be the features that lead to nuanced GxE effects. GxE research may benefit from additional attention to the theorized nature of the candidate environmental variables deployed in GxE research.

To better frame our argument, we consider two stylized patterns of GxE interaction. We emphasize here that these two patterns are not an exhaustive taxonomy of GxE interaction. Rather, they serve as illustrations of the considerations that we encourage. First, consider GxE interactions in which the environmental functions as a “dimmer” on genetic effects. Dimmers, as in switches responsible for dimming or brightening lights, may magnify or constrict genetic effects on an outcome without changing their sign. Investigating dimmer-type GxE may be of high substantive interest in many contexts. For instance, it is of strong practical and theoretical importance to determine whether an educational policy with a robust positive average effect for the population disproportionally benefits children at highest genetic risk or those at lowest genetic risk or has uniform effect across the spectrum of genotypes.

However, as we discuss at greater length in the subsection on coarsened outcome variables, it is also important to be vigilant about the potential for GxE to arise as an artifact of more general effects on the distribution of the observed outcome itself. For instance, suppose the educational policy of interest is associated with an appreciable increase in both the mean and variation of math achievement in the student population. It is then possible that the intervention has increased the effect of the PGS on math achievement (i.e., a positive *b*3 estimate in Eq. [2]) simply as a byproduct of more general increases in variation in math achievement. Because conventional ordinary least squares methods are blind to this type of heteroscedasticity, the concomitant increase in non-PGS variance may go overlooked.

Second, consider GxE interactions in which the environment functions as an image-inverting “lens” on genetic effects. An environment acts as such a lens when the direction of the effect of the PGS differs across the range of that environment. We refer to these environments as lenses based on the optical notion of a lens; in particular, certain glass lenses invert the orientation of objects.^4^ When considering lenses, the relative effect of a given genotype may be positive for a “low” level of the relevant environmental exposure and negative for “high” levels of the exposure, or vice versa. This has led to the hypothesis that what qualifies as a high- or low-risk genotype may depend upon the environmental context (Belsky and Pluess 2009; Ellis et al. 2011; Obradović and Boyce 2009). Note that an environment may function as a lens even when it has a limited main effect.

Researchers frequently conceptualize environments to operate as lenses as a theoretical motivation for doing GxE research, and yet, in practice, many of the environmental measures typically used in GxE studies may be conceptually closer to the dimmer category. Moreover, the selection of PGS effects for examining lens-type GxE may be particularly challenging given that we construct PGSs from GWASs that only include main effects of SNPs (although this is perhaps changing in ways we expand upon below). This limitation may act as a strict limiting factor when it comes to identifying GxE with polygenic scores. A related issue is that, if the environmental context of the participants in the GWAS sample used to construct the PGS is similar to that in the test sample used to estimate GxE, then it is unlikely to include SNPs that demonstrate lens-type patterns, as the main effects of these SNPs will be close to zero.

We can also understand the difference between a dimmer and a lens in terms of their effect on the rank ordering of outcomes. All else being equal, a dimmer is order-preserving; that is, it preserves the order of the genotypes at different levels of the environment. Variation in the dimmer serves to vary the distance, in the outcome metric, between different levels of the PGS but never changes the rank orderings of the levels. In contrast, a lens reverses the order of genotypes; a PGS that predicts an outcome near the top of the distribution at one level of environmental exposure will predict an outcome near the bottom at another level of environmental exposure. Our dimmer/lens typology is similar in many respects to the ordinal/disordinal typology previously suggested (Widaman et al. 2012) but may be a useful conceptual distinction as GxE research becomes more common in the social sciences.

#### Recommendations.

Conceptually, researchers will benefit from being attentive as to the form of GxE they expect; for example, is the candidate environment expected to operate as a lens, as a dimmer, or according to some more complex functional form? In our experience, GxE researchers will tend to observe that environments with large main effects on a phenotype act as dimmers. Such environments will moderate the magnitude of the effect of the polygenic score on the outcome without changing its sign. Although these observations may be of value, they need to be distinguished from the more dramatic patterns of sign reversal of PGS effects in different environments that have received a great deal of conceptual attention. In studies seeking to identify lens-type patterns (Troth et al. 2018), both the genetic and the environmental components are of crucial importance for testing hypotheses in which the environmental context determines whether a given genotype is risky or advantageous.

Analytically, we offer several suggestions that might be of interest in future work. We first emphasize the potential for analyses that take advantage of environmental variation without identifying a specific environmental feature of interest. In situations wherein individuals cluster into some unit, researchers may first want to consider the level of empirical support for GxE based on relatively omnibus measures of the environment. For example, one might test for variation observed in the relationship between phenotype and polygenic score across environmental units (e.g., schools or census tracts); see Trejo et al. (2018) for one such example. Such analyses are informative in that they offer preliminary guidance on whether specific features of the environments deserve additional scrutiny as possible GxE targets.

Yet another approach that researchers might want to consider involves analyses of heritability and genetic correlation as captured by genomic techniques (Grotzinger et al. 2019; Yang et al. 2011). Polygenic scores collapse information from across the genome into a composite designed to predict a specific outcome in a novel data set. Because PGSs are constructed using a large number of GWAS regression weights that themselves are estimated with error, PGS prediction is biased downward in novel samples (and biased upward in the original GWAS samples). In contrast, genomic heritability and genetic correlation estimates are constructed using methods related to mixed effects modeling and are unbiased by measurement error. Such analyses can be used to, for example, study changing patterns of heritability (Tropf et al. 2017) across environments. Although analyses of heritability and genetic correlation do not provide scores for individual participants (because they estimate random effects to represent population variation, rather than individual estimates [de Vlaming et al. 2017]), they can still offer information about the way that genotypes are related to phenotypes.

We also note the increase in methodologies focused on identifying genetic variants that are associated with the amount of variation in the outcome rather than strictly the level (Conley et al. 2018; Wang et al. 2019; Yang et al. 2012; Young, Wauthier, and Donnelly 2018). Such approaches are generating data that may be useful in future GxE work. A natural question to ask of the genetic variants identified in such studies is whether environments interact with such variants to further modulate variation in the outcome. Although such approaches will presumably also involve novel methodological challenges, they are an exciting new resource that could be used to study gene–environment interplay.

### Coarsened Outcome Variables

#### The problem.

Characteristics of the distribution of *ϒ* may have crucial implications for conducting GxE studies. When *ϒ* is a discrete outcome coarsened from an underlying continuous variable, researchers encounter an opportunity to mis-interpret affirmative findings of GxE. For simplicity of exposition, we focus on the simplest case where *ϒ* is dichotomous (though the phenomenon extends to coarsened variables that take more than two values). Suppose a dichotomous outcome *ϒ* is a coarsened version of some continuously varying latent indicator ϒ_*_ (so *ϒ* = 1 if ϒ_*_ > *λ* for some scalar *λ* and 0 otherwise). For example, *ϒ* might be obesity or college completion (in which case ϒ_*_ would be body mass index or years of schooling, respectively). Suppose we estimate Equation (2) with ordinary least squares using ϒ_*_ instead of *ϒ* and yield a nonzero and statistically significant *b*3. How should we interpret such a finding? One possibility is that a finding of GxE suggests differences in the slope of association between *G* and ϒ_*_. This, we argue, is what researchers generally have in mind when conducting studies testing for GxE. However, a second possibility is that a purely environmental shock may shift the intercept of the association line between *G* and Y_*_, thus resulting in a GxE finding (i.e., a nonzero and statistically significant *b*3) with different interpretation.

**Figure 1:**
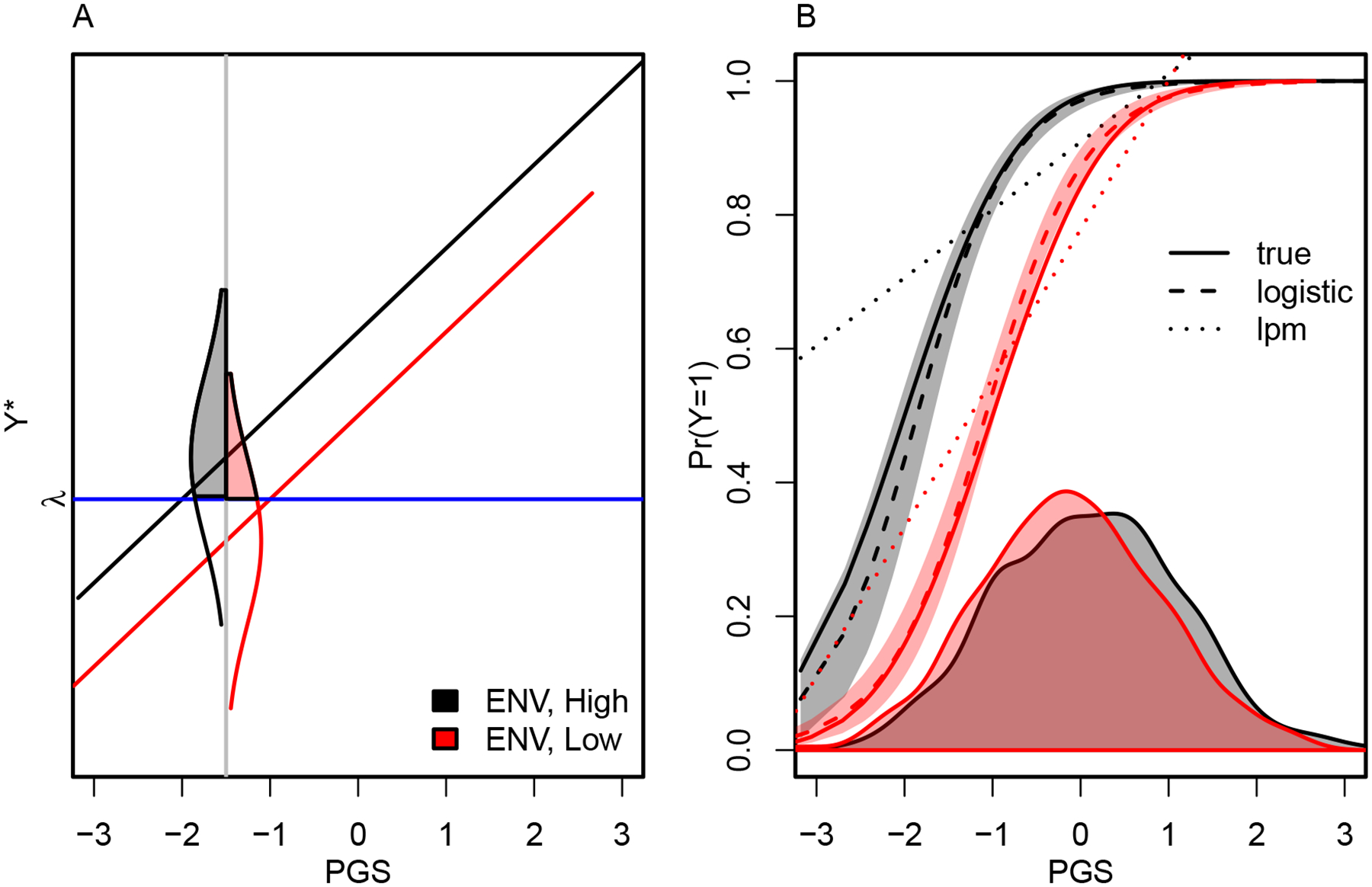
The challenge of studying GxE when using dichotomous outcomes. (*A*) True association between PGS and continuously varying outcome ϒ_*_. Densities show distributions above horizontal blue line for those in high and low environments. (*B*) True associations and those estimated using either a logistic regression model or a linear probability model when ϒ_*_ has been dichotomized prior to analysis (*ϒ* = 1 when Y_*_ > *λ*). The linear probability model (lpm) produces the misimpression of GxE (nonparallel regression lines). The logistic regression model does not suffer from this bias but may still suffer from large standard errors and low power when we observe low variability in the dichotomous *ϒ* variable in one of the environments.

We illustrate the basic problem in Figure 1. When we examine relationships between PGS and outcome in the context of the continuously measured version—ϒ_*_ in Figure 1A—we observe a constant linear association with genotype across two environments. However, when we observe a dichotomized version of the outcome—*ϒ* in Figure 1B—we have a relationship that is more challenging to interpret. In particular, Figure 1B suggests GxE when a linear probability model is used (i.e., the dotted curves are not parallel). In contrast, when a logistic regression model is used, we obtain unbiased estimates of GxE (i.e., *b*3) but they may suffer from low power (and large confidence intervals) due to low variability in the dichotomized outcome at some regions of the environmental measure. This problem may be even more severe when gene–environment correlation results in a large shift in the distributions of the PGS along the range of the environmental measure.

Findings such as those in Figure 1B are worth noting, and they may be highly relevant in cases where the continuous ϒ_*_ is of less interest than the dichotomized *ϒ* (e.g., college completion may well matter more than years of schooling) or when ϒ_*_ is latent. However, we also need not confuse matters by misunderstanding the nature of the associations in question. If findings are driven by differences in intercepts and relatively consistent slopes, as in Figure 1A, this is important information to report. We expect that GxE research will benefit from distinguishing between these two possibilities; see also our discussion of this issue in an empirical context elsewhere (Trejo et al. 2018).^5^

#### Recommendations.

When research uses coarsened outcome variables due to substantive interest in the coarsened outcome themselves (e.g., credentials, obesity indicators), sensitivity analyses that probe the issue considered here based on the underlying (noncoarsened) variable are essential. Such analyses will help to better contextualize findings from coarsened variables. In analysis of binary outcomes for which no underlying continuous variable is available (i.e., case-control status), utilization of multiple methods, such as both logistic and linear probability models, may be used to probe for sensitivity of the results to the functional form of the model. This will be especially important when the environment is itself nontrivially correlated with the outcome under study.

Although we do not focus here on coarsened outcomes that are nonbinary (e.g., ordered categorical, nominal, or censored/truncated outcomes), we note that many of the concerns raised here would be of relevance in those cases as well. At a minimum, sensitivity analyses probing the persistence of findings across a range of model specifications may be valuable. For example, in an analysis of the highest math course taken by high schools students (Harden et al. 2020), a variety of models—cumulative link, adjacent-category logit, locally estimated scatterplot smoothing (LOESS) based on dichotomizations—were used in an attempt to interrogate potential differences in course as a function of genotype when stratified by school socioeconomic status. GxE analysis in the context of such coarsened outcomes is likely to be challenging; future work describing methodological best practices in this domain would be welcome.

### Measurement Error

#### The problem.

Measurement error acts both to bias associated parameter estimates toward zero (Hutcheon, Chiolero, and Hanley 2010) and to distort power calculations. In the specific context of GxE studies, there are several concerns. Measurement error exists in both the operationalized PGS and ENV variables of Equation (2). Measurement error in *G*, which results from imprecise estimates of the GWAS betas used to construct the PGS, has received some attention (Conley et al. 2016; DiPrete, Burik, and Koellinger 2018; Tucker-Drob 2017). However, less attention has been paid to measurement error in *E*. Homoscedastic measurement error in *E* has implications for power (matters may be further complicated in the presence of nonhomoscedastic measurement error, but we focus on the simpler case here).

**Figure 2:**
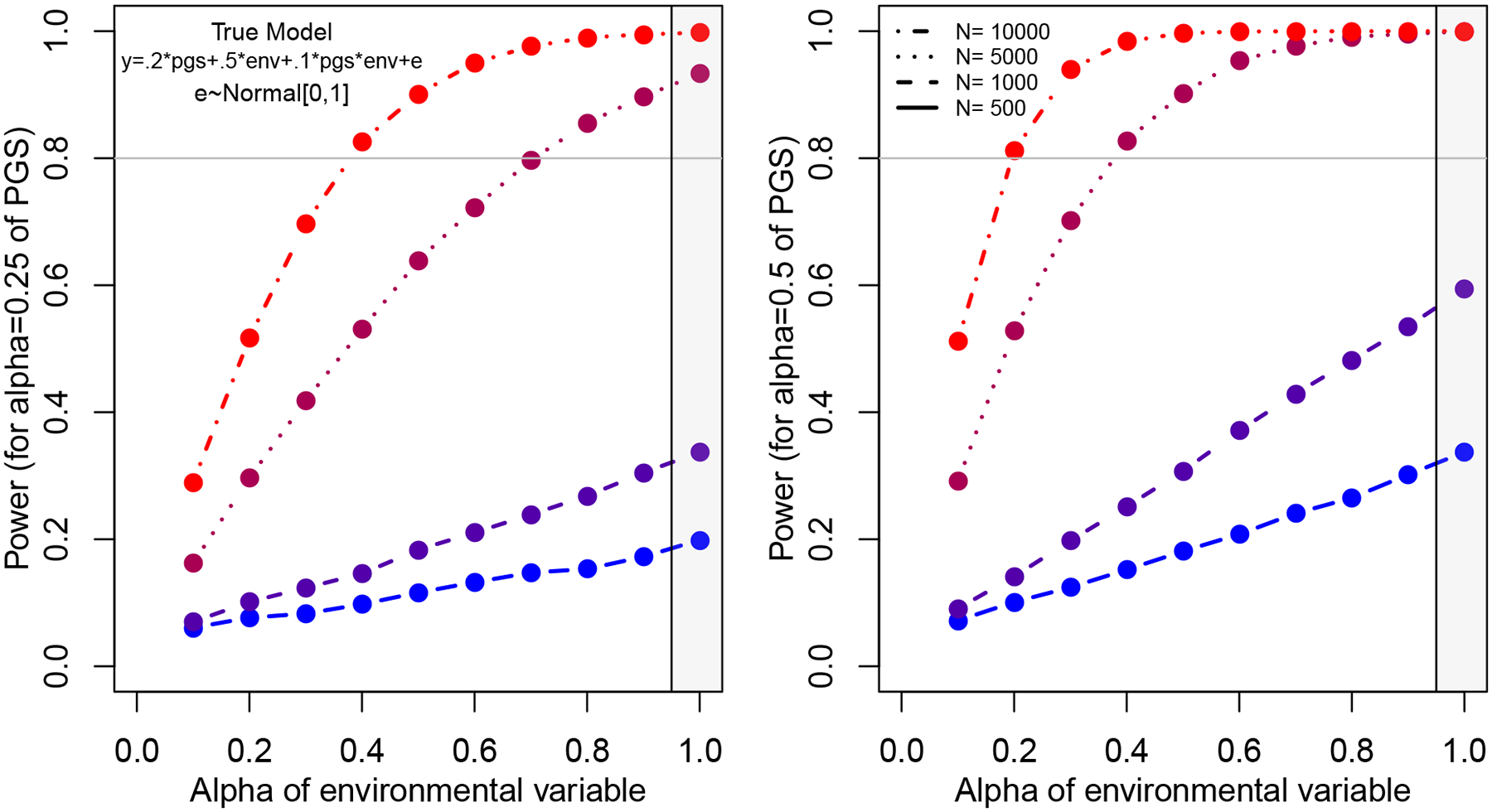
Reduction in power as a function of measurement error in both PGS and ENV. Left and right panels focus on relatively low (alpha = 0.25) and high (alpha = 0.5) reliability polygenic scores. Data-generating equation is shown in left-hand panel.

Figure 2 is a simple illustration of this via a simulation study.^6^ We assume that we measure both the PGS and the target environmental variable with error. We focus on variation in the reliability of the environmental measure (the *x* axis) and choose two levels of reliability (which we index as alpha) of 0.25 (on left) and 0.5 (on right) for the PGS; we view these reliabilities as representative of relatively weak and relatively strong polygenic scores given existing GWAS. The main takeaway is that ignoring measurement error with respect to the environment inevitably leads to inflated power calculations.

Let us first focus our attention on a PGS with relatively high reliability by current PGS standards (alpha = 0.5) in the case where we have 1,000 respondents. We first assume that there is no error in our environmental measure (region emphasized in gray rectangle). In such a case, power is below standard levels of acceptability (power = 0.8). As the reliability of our environmental measure declines, however, power becomes increasingly poor. Even when the environment is measured with decent reliability (alpha = 0.7), power is greatly reduced (power = 0.4). In the case where the PGS is of lower reliability, power is even worse (power = 0.2 for an environmental measure of reliability alpha = 0.7). When the PGS is measured with substantial error (alpha = 0.25), even relatively large samples (when considering population-based studies) of *N* = 10,000 will suffer from power limitations when the environment is also measured with substantial error. These calculations are based upon a toy model that might not be relevant in all cases, but given that interaction studies are power-hungry even without considering measurement error (McClelland and Judd 1993), our model emphasizes the need to carefully consider whether one has reasonable power before conducting GxE studies.

#### Recommendations.

We recommend that power analyses be the norm (and not the exception) in GxE research. Traditional power analyses are used to inform key design features, such as the sample size, prior to the implementation of a study. In contrast, power analyses of the type considered here offer information about the power of a study design given existing data (e.g., the sample sizes available from large longitudinal studies such as the HRS and Add Health) and key assumptions about the relevant parameters. In particular, power analyses specifically designed to offer insights into the level of power available given measurement error in both the polygenic score and the environment would be valuable additions when planning analyses of data that are already available. As Figure 2 illustrates, a failure to consider measurement error can lead to inflated estimates of power. Even for samples of several thousand, GxE analyses will be weakly powered absent highly penetrant genetic predictors or environments measured with little noise. Such power analyses are not cure-alls; rather, they hopefully help researchers to better understand the limitations that they face—specifically, the likelihood of observing false positives—in a given context.

### Sample Selection Processes and Internal and External Validity

#### The problem.

Selection processes complicate inference in observational settings in a number of ways, and studies of GxE are no exception. An often-underappreciated point is that sample selection issues threaten both external and internal validity. We discuss several (potentially overlapping) types of selection that are particularly relevant for GxE research. These sample selection processes limit the population to which GxE findings can be generalized and may lead to spurious results via collider bias (Elwert and Winship 2014). Notably, sample selection may pose a threat both in the discovery GWAS used to identify the betas needed to construct a PGS and in the prediction sample in which the PGS is actually constructed and used to estimate GxE.

We begin with mortality selection. Such selection occurs when a nonrandom subset is lost to mortality and therefore not observed. In studies of older respondents (e.g., the HRS), mortality selection tends to make the resulting sample “healthier, wealthier, and wiser” (Zajacova and Burgard 2013). Mortality selection is especially relevant to GxE research because genotyping is a relatively recent technology; participants in longstanding studies needed to survive long enough to make it into the genotyped subsample. Indeed, there is evidence to suggest that GxE findings may be sensitive to the presence of mortality selection (Domingue et al. 2017). When studying health-related traits, especially in older populations, we need to consider mortality selection’s role in shaping findings (Oliynyk 2019). In scenarios wherein mortality can be readily modeled with existing data, one possible analytic solution is to use inverse probability weighting (van der Wal and Geskus 2011) to correct for the role of mortality selection. A related issue is that individuals with certain genetic profiles—for example, those with high genetic liabilities for schizophrenia—may be underrepresented in various data sources (Martin et al. 2016; Meisner, Kundu, and Chatterjee 2019; Pirastu et al. 2020; Taylor et al. 2018). Such selection can also lead to issues of both bias and generalizability in subsequent studies.

A second issue is that demographic factors play a role in who gets included in genetic studies. This, in turn, has implications with respect to the populations to which results using genetic subsamples may generalize. Of particular note is the massive overrepresentation of European-descent individuals in both GWAS (Mills and Rahal 2019) and PGS (Duncan et al. 2019) studies. This problem is due to several factors, including both the overrepresentation of European-descent individuals in genetic studies and the fact that differences in linkage disequilibrium across groups leads to the GWAS findings performing better in the (predominantly European) samples from which they are derived. Efforts (Mills and Rahal 2020) are underway to monitor (with the hope of then remedying) this problem. In the meantime, researchers have noted that adoption of polygenic scores in precision medicine may exacerbate preexisting health disparities (Martin et al. 2019). A focus on homogeneous samples may lead to issues in GxE if it either severely constricts the relevant artificial variance or even potentially undermines the theoretical motivation suggesting a particular research question (which may necessitate a more diverse sample). In any event, equity concerns need to be in the foreground of genetics research; GxE is no exception.

These selection problems offer both internal and external validity threats to GxE studies that are important to consider carefully. An additional concern is that nonrandom selection into the analytic samples used in empirical studies may lead to reduced environmental variation further challenging attempts to make accurate inferences regarding GxE. As an illustration, we consider two key adolescent environments—the socioeconomic circumstances of home (Belsky et al. 2018) and the disadvantage of one’s residential neighborhood (Belsky et al. 2019), both from Wave I of Add Health (Harris et al. 2019)—that may be of interest. As a function of the way the analytic sample becomes a selected portion of the full sample, we observe a decrease in environmental variance. These decreases will lead to even further reductions in our power to detect GxE effects; in particular, power analyses motivated by environmental variation observed in the full sample are likely to overstate true power given that empirical work will then take place with reduced environmental variation. Beyond power concerns, such selection can lead to a reduction in density in certain regions of the distribution of the measured environment that will increase the challenge of identifying the relevant functional form in that region.

#### Recommendations.

**Figure 3:**
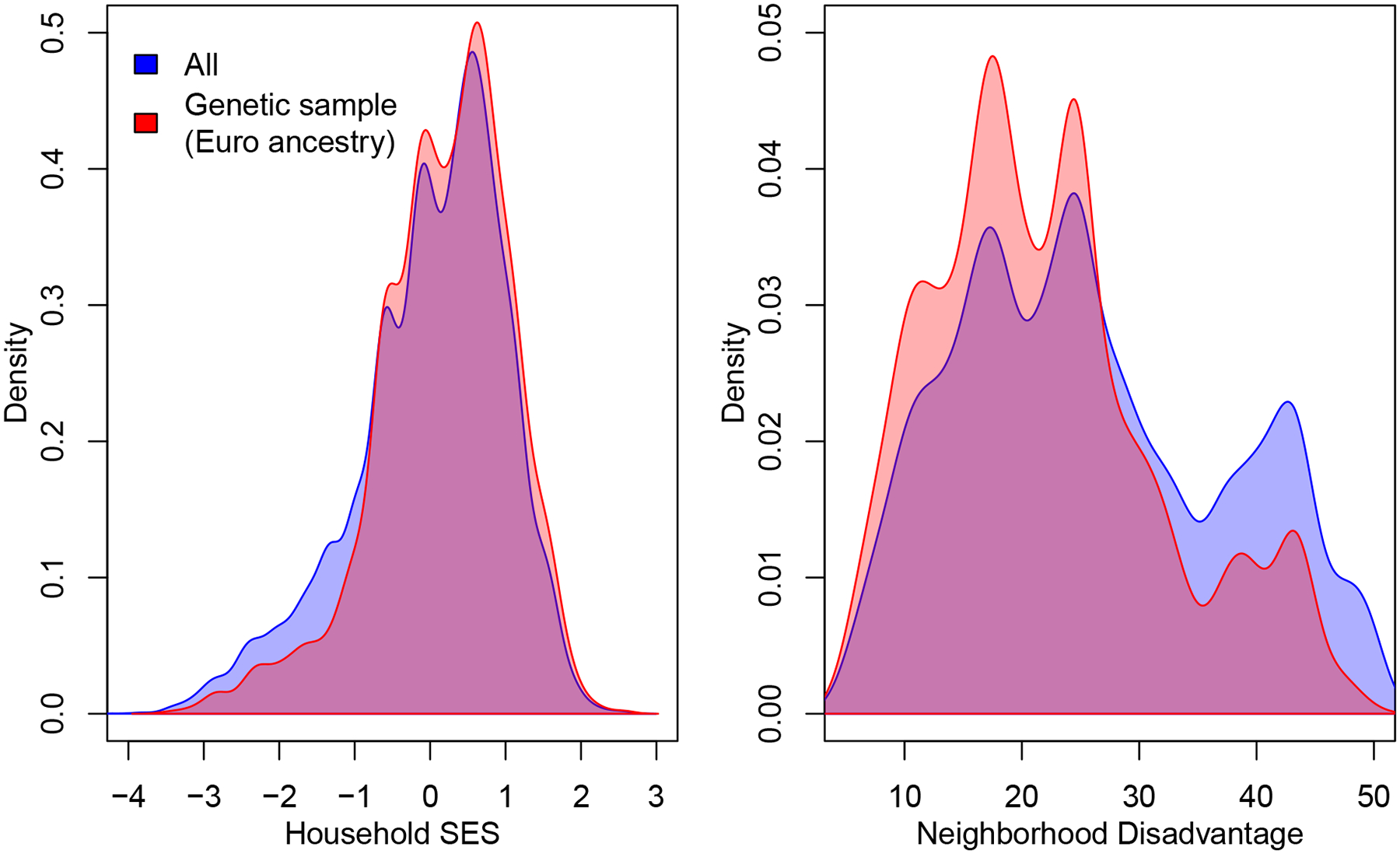
Distributions of two key environmental variables (household socioeconomic status [SES] and neighborhood disadvantage) taken from Wave I of Add Health (Harris et al. 2019). Note the reduction in variation of the distribution for the analytic sample (in red) versus that of the full sample (in blue). Reductions in the standard deviation are 11 percent for SES and 14 percent for neighborhood disadvantage; in variance terms, the reductions are 20 percent and 26 percent, respectively.

Issues concerning selection require careful attention. Figure 3 suggests that they may have implications that need to be accounted for in other aspects of study design (i.e., are power analyses based on the appropriate quantities?). We further suggest two ways that research may approach these issues. First, the selection issues discussed here have implications for generalizability. Some forms of this problem are obvious. Given, for example, the problems of analysis in ancestrally heterogeneous samples and the subsequent work on samples of relatively limited genetic diversity, it would be imprudent to interpret GxE findings from such a study as applying in the broader population containing a fuller spectrum of genetic diversity. But it may also be the case that selection introduces other factors that limit generalizability. For example, long-lived smokers may be quite different from the general population (Levine and Crimmins 2014); inference based on such samples may be misleading.

Second, on the analytic side, attempts to model the relevant selection processes may lead to direct insights into the degree of generalizability of patterns. For example, researchers may examine how results change when using formal techniques that correct for selection (e.g., inverse probability weighting [Cole and Hernán 2008]). Even less comprehensive analyses of selection processes may lead to insights about the nature of the analytic sample and offer guides to generalization that researchers can communicate alongside the relevant empirical results.

## Conclusion

GxE characterizes both the environmental contingency of genetically linked processes and the genetic contingency of environmentally linked processes. In our view, GxE studies involving human behavior and polygenic scores may offer valuable insights but are also at risk of repeating many of the mistakes made by previous eras of research (e.g., the candidate gene era). Our goal has been to emphasize the need for careful thinking about the rationale and methods underlying investigations of GxE.

In particular, we highlighted four issues—selection of the relevant environment, analysis of coarsened outcomes, the role of measurement error, and issues of sample selection—that deserve additional scrutiny in future research. We also attempted to offer recommendations for beginning to address each problem. We readily acknowledge that ours are a relatively modest set of recommendations that will not fully resolve the vast range of analytic and inferential challenges associated with GxE research with PGSs.

An overarching goal of research examining the combined genetic and environmental contributions to human behavior is to help construct *useful* models of human behavior. In our view, *useful* models avoid unnecessary complexity when accounting for messy data. At its best, GxE research can help inform the construction of such models by parsimoniously showcasing complexities from empirical reality that need to be accounted for. For instance, GxE research can help reveal important heterogeneity in developmental processes, treatment responses, and policy effects. To be informative, however, we must exercise care. Otherwise, GxE research threatens to introduce confusion into the already challenging landscape of social and behavioral science research.

## Notes

1. Exactly how predictive a PGS is of a given trait depends on both on the trait’s heritability and the sample size of the GWAS used to derive the effect size estimates; see Figure 2 of Harden and Koellinger (2020).
2. We note that one could alternatively discuss environmental effects differing as a function of genetics; we utilize the original formulation in this article but note that the latter may occasionally be the more germane.
3. In practice, polygenic scores may contain information on correlated nongenetic factors (e.g., population stratification and dynastic effects like genetic nurture) in addition to true genetic risk (Morris et al. 2020).
4. Specifically, convex lenses have such image-inverting properties. Here we use “lens” as shorthand for convex lens but note that concave lenses do not have this property.
5. For a similar observation in a different context, see https://twitter.com/Joni_Coleman/status/1220332653599186946?s=20.
6. Code available here: https://gist.github.com/ben-domingue/6f14e3c4532ecb62df5f6e0c44c60411.

## References

[R1] Barr Peter B., Silberg Judy, Dick Danielle M., and Maes Hermine H.. 2018. “Childhood Socioeconomic Status and Longitudinal Patterns of Alcohol Problems: Variation across Etiological Pathways in Genetic Risk.” Social Science & Medicine 209:51–58. 10.1016/j.socscimed.2018.05.027. 29793164 PMC5997543

[R2] Belsky Daniel W., Caspi Avshalom, Arseneault Louise, Corcoran David L., Domingue Benjamin W., Harris Kathleen Mullan, Houts Renate M., Mill Jonathan S., Moffitt Terrie E., Prinz Joseph, Sugden Karen, Wertz Jasmin, Williams Benjamin, and Odgers Candice L.. 2019. “Genetics and the Geography of Health, Behaviour and Attainment.” Nature Human Behaviour 3:576–86. 10.1038/s41562-019-0562-1. PMC6565482 30962612

[R3] Belsky Daniel W., Domingue Benjamin W., Wedow Robbee, Arseneault Louise, Boardman Jason D., Caspi Avshalom, Conley Dalton C., Fletcher Jason M., Freese Jeremy, Herd Pamela, Moffitt Terrie E., Poulton Richie, Sicinski Kamil, Wertz Jasmin, and Harris Kathleen Mullan. 2018. “Genetic Analysis of Social-Class Mobility in Five Longitudinal Studies.” Proceedings of the National Academy of Sciences 115(31):E7275–84. 10.1073/pnas.1801238115. PMC6077729 29987013

[R4] Belsky Daniel W., and Harden K. Paige. 2019. “Phenotypic Annotation: Using Polygenic Scores to Translate Discoveries from Genome-Wide Association Studies from the Top Down.” Current Directions in Psychological Science 28(1):82–90. 10.1177/0963721418807729. PMC11086979 38736689

[R5] Belsky Jay, and Pluess Michael. 2009. “Beyond Diathesis Stress: Differential Susceptibility to Environmental Influences.” Psychological Bulletin 135(6):885–908. 10.1037/a0017376. 19883141

[R6] Boardman Jason D., Barnes Lisa L., Wilson Robert S., Evans Denis A., and Mendes de Leon Carlos F.. 2012. “Social Disorder, APOE-E4 Genotype, and Change in Cognitive Function among Older Adults Living in Chicago.” Social Science & Medicine 74(10):1584–90. 10.1016/j.socscimed.2012.02.012. 22465377 PMC3597347

[R7] Boardman Jason D., Daw Jonathan, and Freese Jeremy. 2013. “Defining the Environment in Gene–Environment Research: Lessons from Social Epidemiology.” American Journal of Public Health 103(S1):S64–72. 10.2105/AJPH.2013.301355. 23927514 PMC3786759

[R8] Boyle Evan A., Li Yang I., and Pritchard Jonathan K.. 2017. “An Expanded View of Complex Traits: From Polygenic to Omnigenic.” Cell 169(7):1177–86. 10.1016/j.cell.2017.05.038. 28622505 PMC5536862

[R9] Braudt David B., and Harris Kathleen Mullan. 2018. “Polygenic Scores (PGSs) in the National Longitudinal Study of Adolescent to Adult Health (Add Health)–Release 1.” Carolina Population Center, University of North Carolina at Chapel Hill. 10.17615/C6M372.

[R10] Briley Daniel A., Livengood Jonathan, Derringer Jaime, Tucker-Drob Elliot M., Fraley R. Chris, and Roberts Brent W.. 2019. “Interpreting Behavior Genetic Models: Seven Developmental Processes to Understand.” Behavior Genetics 49(2):196–210. 10.1007/s10519-018-9939-6. 30467668 PMC6904232

[R11] Cesarini David, and Visscher Peter M.. 2017. “Genetics and Educational Attainment.” NPJ Science of Learning 2(1):4. 10.1038/s41539-017-0005-6. 30631451 PMC6220209

[R12] Chabris Christopher F., Lee James J., Cesarini David, Benjamin Daniel J., and Laibson David I.. 2015. “The Fourth Law of Behavior Genetics.” Current Directions in Psychological Science 24(4):304–12. 10.1177/0963721415580430. 26556960 PMC4635473

[R13] Cole Stephen R., and Hernán Miguel A.. 2008. “Constructing Inverse Probability Weights for Marginal Structural Models.” American Journal of Epidemiology 168(6):656–64. 10.1093/aje/kwn164. 18682488 PMC2732954

[R14] Conley Dalton, and Fletcher Jason. 2017. The Genome Factor: What the Social Genomics Revolution Reveals about Ourselves, Our History, and the Future. Princeton: Princeton University Press. 10.1515/9781400883240.

[R15] Conley Dalton, Johnson Rebecca, Domingue Ben, Dawes Christopher, Boardman Jason, and Siegal Mark. 2018. “A Sibling Method for Identifying VQTLs.” PloS One 13(4):e0194541. 10.1371/journal.pone.0194541. 29617452 PMC5884517

[R16] Conley Dalton, Laidley Thomas M., Boardman Jason D., and Domingue Benjamin W.. 2016. “Changing Polygenic Penetrance on Phenotypes in the 20th Century among Adults in the US Population.” Scientific Reports 6:30348. 10.1038/srep30348. 27456657 PMC4960614

[R17] de Vlaming Ronald, Johannesson Magnus, Magnusson Patrik K. E., Ikram M. Arfan, and Visscher Peter M.. 2017. “Equivalence of LD-Score Regression and Individual-Level-Data Methods.” bioRxiv. Preprint, submitted October 31. https://www.biorxiv.org/content/10.1101/211821v1.

[R18] DiPrete Thomas A., Burik Casper A. P., and Koellinger Philipp D.. 2018. “Genetic Instrumental Variable Regression: Explaining Socioeconomic and Health Outcomes in Nonexperimental Data.” Proceedings of the National Academy of Sciences 115(22):E4970–79. 10.1073/pnas.1707388115. PMC5984483 29686100

[R19] Domingue Benjamin W., Belsky Daniel W., Harrati Amal, Conley Dalton, Weir David, and Boardman Jason. 2017. “Mortality Selection in a Genetic Sample and Implications for Association Studies.” International Journal of Epidemiology 46(4):1285–94. 10.1093/ije/dyx041. 28402496 PMC5837559

[R20] Dudbridge Frank. 2013. “Power and Predictive Accuracy of Polygenic Risk Scores.” PLoS Genetics 9(3):e1003348. 10.1371/journal.pgen.1003348. 23555274 PMC3605113

[R21] Dudbridge Frank. 2016. “Polygenic Epidemiology.” Genetic Epidemiology 40(4):268–72. 10.1002/gepi.21966. 27061411 PMC4982028

[R22] Dudbridge Frank, and Fletcher Olivia. 2014. “Gene-Environment Dependence Creates Spurious Gene-Environment Interaction.” American Journal of Human Genetics 95(3):301–7. 10.1016/j.ajhg.2014.07.014. 25152454 PMC4157149

[R23] Duncan Greg J., and Magnuson Katherine. 2012. “Socioeconomic Status and Cognitive Functioning: Moving from Correlation to Causation.” Wiley Interdisciplinary Reviews: Cognitive Science 3(3):377–86. 10.1002/wcs.1176. 26301469

[R24] Duncan L, Shen H, Gelaye B, Meijsen J, Ressler K, Feldman M, Peterson R, and Domingue B. 2019. “Analysis of Polygenic Risk Score Usage and Performance in Diverse Human Populations.” Nature Communications 10(1):3328. 10.1038/s41467-019-11112-0. PMC6658471 31346163

[R25] Duncan Laramie E., and Keller Matthew C.. 2011. “A Critical Review of the First 10 Years of Candidate Gene-by-Environment Interaction Research in Psychiatry.” American Journal of Psychiatry 168(10):1041–49. 10.1176/appi.ajp.2011.11020191. 21890791 PMC3222234

[R26] Ellis Bruce J., Boyce W. Thomas, Belsky Jay, Bakermans-Kranenburg Marian J., and Van Ijzendoorn Marinus H.. 2011. “Differential Susceptibility to the Environment: An Evolutionary–Neurodevelopmental Theory.” Development and Psychopathology 23(01):7–28. 10.1017/S0954579410000611. 21262036

[R27] Elwert Felix, and Winship Christopher. 2014. “Endogenous Selection Bias: The Problem of Conditioning on a Collider Variable.” Annual Review of Sociology 40:31–53. 10.1146/annurev-soc-071913-043455. PMC6089543 30111904

[R28] Feldman MW, and Lewontin RC. 1975. “The Heritability Hang-Up.” Science 190(4220):1163–68. 10.1126/science.1198102. 1198102

[R29] Fletcher Jason M., and Conley Dalton. 2013. “The Challenge of Causal Inference in Gene–Environment Interaction Research: Leveraging Research Designs from the Social Sciences.” American Journal of Public Health 103(S1):S42–45. 10.2105/AJPH.2013.301290. 23927518 PMC3786757

[R30] Freese Jeremy. 2018. “The Arrival of Social Science Genomics.” Contemporary Sociology 47(5):524–36. 10.1177/0094306118792214a.

[R31] Gould Karen L., Coventry William L., Olson Richard K., and Byrne Brian. 2018. “Gene-Environment Interactions in ADHD: The Roles of SES and Chaos.” Journal of Abnormal Child Psychology 46(2):251–263. 10.1007/s10802-017-0268-7. 28283857

[R32] Grotzinger Andrew D., Rhemtulla Mijke, Ronald de Vlaming Stuart J. Ritchie, Mallard Travis T., Hill W. David, Ip Hill F., Marioni Riccardo E., McIntosh Andrew M., Deary Ian J., Koellinger Philipp D., Harden K. Paige, Nivard Michel G., and Tucker-Drob Elliot M.. 2019. “Genomic Structural Equation Modelling Provides Insights into the Multivariate Genetic Architecture of Complex Traits.” Nature Human Behaviour 3(5):513–25. 10.1038/s41562-019-0566-x. PMC6520146 30962613

[R33] Harden K. Paige, Domingue Benjamin W., Belsky Daniel W., Boardman Jason D., Crosnoe Robert, Malanchini Margherita, Nivard Michel, Tucker-Drob Elliot M., and Harris Kathleen Mullan. 2020. “Genetic Associations with Mathematics Tracking and Persistence in Secondary School.” NPJ Science of Learning 5(1):1–8. 10.1038/s41539-020-0060-2. 32047651 PMC7002519

[R34] Harden K. Paige, and Koellinger Philipp D.. 2020. “Using Genetics for Social Science.” Nature Human Behaviour 4:567. 10.1038/s41562-020-0862-5. PMC8240138 32393836

[R35] Harris Kathleen Mullan, Halpern Carolyn Tucker, Whitsel Eric A., Hussey Jon M., Killeya-Jones Ley A., Tabor Joyce, and Dean Sarah C.. 2019. “Cohort Profile: The National Longitudinal Study of Adolescent to Adult Health (Add Health).” International Journal of Epidemiology 48(5):1415. 10.1093/ije/dyz115. 31257425 PMC6857761

[R36] Hutcheon Jennifer A., Chiolero Arnaud, and Hanley James A.. 2010. “Random Measurement Error and Regression Dilution Bias.” BMJ 340:c2289. 10.1136/bmj.c2289. 20573762

[R37] Jaffee Sara R., and Price Thomas S.. 2007. “Gene–Environment Correlations: A Review of the Evidence and Implications for Prevention of Mental Illness.” Molecular Psychiatry 12(5):432–42. 10.1038/sj.mp.4001950. 17453060 PMC3703541

[R38] Johnson Sara B., Riis Jenna L., and Noble Kimberly G.. 2016. “State of the Art Review: Poverty and the Developing Brain.” Pediatrics 137(4):e20153075. 10.1542/peds.2015-3075. 26952506 PMC4811314

[R39] Keller Matthew C. 2014. “Gene × Environment Interaction Studies Have Not Properly Controlled for Potential Confounders: The Problem and the (Simple) Solution.” Biological Psychiatry 75(1):18–24. 10.1016/j.biopsych.2013.09.006. 24135711 PMC3859520

[R40] Lambert Samuel A., Gil Laurent, Jupp Simon, Ritchie Scott C., Xu Yu, Buniello Annalisa, Abraham Gad, Chapman Michael, Parkinson Helen, Danesh John, MacArthur Jacqueline A., and Inouye Michael. 2020. “The Polygenic Score Catalog: An Open Database for Reproducibility and Systematic Evaluation.” medRxiv. Preprint, submitted May 23. https://www.medrxiv.org/content/10.1101/2020.05.20.20108217v1. 10.1038/s41588-021-00783-5 PMC11165303 33692568

[R41] Lee James J., Wedow Robbee, Okbay Aysu, Kong Edward, Maghzian Omeed, Zacher Meghan, Nguyen-Viet Tuan Anh, Bowers Peter, Sidorenko Julia, Linnér Richard Karlsson, Fontana Mark Alan, Kundu Tushar, Lee Chanwook, Li Hui, Li Ruoxi, Royer Rebecca, Timshel Pascal N., Walters Raymond K., Willoughby Emily A., Yengo Loïc, Alver Maris, Bao Yanchun, Clark David W., Day Felix R., Furlotte Nicholas A., Joshi Peter K., Kemper Kathryn E., Kleinman Aaron, Langenberg Claudia, Mägi Reedik, Trampush Joey W., Verma Shefali Setia, Wu Yang, Lam Max, Zhao Jing Hua, Zheng Zhili, Boardman Jason D., Campbell Harry, Freese Jeremy, Harris Kathleen Mullan, Hayward Caroline, Herd Pamela, Kumari Meena, Lencz Todd, Luan Jian’an, Malhotra Anil K., Metspalu Andres, Milani Lili, Ong Ken K., Perry John R. B., Porteous David J., Ritchie Marylyn D., Smart Melissa C., Smith Blair H., Tung Joyce Y., Wareham Nicholas J., Wilson James F., Beauchamp Jonathan P., Conley Dalton C., Esko Tõnu, Lehrer Steven F., Magnusson Patrik K. E., Oskarsson Sven, Pers Tune H., Robinson Matthew R., Thom Kevin, Watson Chelsea, Chabris Christopher F., Meyer Michelle N., Laibson David I., Yang Jian, Johannesson Magnus, Koellinger Philipp D., Turley Patrick, Visscher Peter M., Benjamin Daniel J., and Cesarini David. 2018. “Gene Discovery and Polygenic Prediction from a Genome-Wide Association Study of Educational Attainment in 1.1 Million Individuals.” Nature Genetics 50(8):1112–21. 10.1038/s41588-018-0147-3. 30038396 PMC6393768

[R42] Levine Morgan, and Crimmins Eileen. 2014. “Not All Smokers Die Young: A Model for Hidden Heterogeneity within the Human Population.” PloS One 9(2):e87403. 10.1371/journal.pone.0087403. 24520332 PMC3919713

[R43] Lubinski David, and Humphreys Lloyd G.. 1990. “Assessing Spurious ‘Moderator Effects’: Illustrated Substantively with the Hypothesized (‘Synergistic’) Relation between Spatial and Mathematical Ability.” Psychological Bulletin 107(3):385–93. 10.1037/0033-2909.107.3.385. 2349320

[R44] MacCallum Robert C., and Mar Corinne M.. 1995. “Distinguishing between Moderator and Quadratic Effects in Multiple Regression.” Psychological Bulletin 118(3):405–21. 10.1037/0033-2909.118.3.405.

[R45] Martin Alicia R., Kanai Masahiro, Kamatani Yoichiro, Okada Yukinori, Neale Benjamin M., and Daly Mark J.. 2019. “Clinical Use of Current Polygenic Risk Scores May Exacerbate Health Disparities.” Nature Genetics 51(4):584–91. 10.1038/s41588-019-0379-x. 30926966 PMC6563838

[R46] Martin Joanna, Tilling Kate, Hubbard Leon, Stergiakouli Evie, Thapar Anita, Smith George Davey, O’Donovan Michael C., and Zammit Stanley. 2016. “Association of Genetic Risk for Schizophrenia with Nonparticipation over Time in a Population-Based Cohort Study.” American Journal of Epidemiology 183(12):1149–58. 10.1093/aje/kww009. 27188935 PMC4908211

[R47] McClelland Gary H., and Judd Charles M.. 1993. “Statistical Difficulties of Detecting Interactions and Moderator Effects.” Psychological Bulletin 114(2):376–90. 10.1037/0033-2909.114.2.376. 8416037

[R48] Meisner Allison, Kundu Prosenjit, and Chatterjee Nilanjan. 2019. “Case-Only Analysis of Gene-Environment Interactions Using Polygenic Risk Scores.” American Journal of Epidemiology 188(11):2013–20. 10.1093/aje/kwz175. 31429870

[R49] Mills Melinda C., and Rahal Charles. 2019. “A Scientometric Review of Genome-Wide Association Studies.” Communications Biology 2(1):9. 10.1038/s42003-018-0261-x. 30623105 PMC6323052

[R50] Mills Melinda C., and Rahal Charles. 2020. “The GWAS Diversity Monitor Tracks Diversity by Disease in Real Time.” Nature Genetics 52(3):242–43. 10.1038/s41588-020-0580-y. 32139905

[R51] Mills Melinda C., and Tropf Felix C.. 2020. “Sociology, Genetics, and the Coming of Age of Sociogenomics.” Annual Review of Sociology 46:553–81. 10.1146/annurev-soc-121919-054756.

[R52] Morris Tim T., Davies Neil M., Hemani Gibran, and Smith George Davey. 2020. “Population Phenomena Inflate Genetic Associations of Complex Social Traits.” Science Advances 6(16):eaay0328. 10.1126/sciadv.aay0328. 32426451 PMC7159920

[R53] Mostafavi Hakhamanesh, Harpak Arbel, Agarwal Ipsita, Conley Dalton, Pritchard Jonathan K., and Przeworski Molly. 2020. “Variable Prediction Accuracy of Polygenic Scores within an Ancestry Group.” eLife 9:e48376. 10.7554/eLife.48376. 31999256 PMC7067566

[R54] Musci Rashelle J., Bettencourt Amie F., Sisto Danielle, Maher Brion, Masyn Katherine, and Ialongo Nicholas S.. 2019. “Violence Exposure in an Urban City: A GxE Interaction with Aggressive and Impulsive Behaviors.” Journal of Child Psychology and Psychiatry 60(1):72–81. 10.1111/jcpp.12966. 30159911 PMC6392042

[R55] Obradović Jelena, and Boyce W. Thomas. 2009. “Individual Differences in Behavioral, Physiological, and Genetic Sensitivities to Contexts: Implications for Development and Adaptation.” Developmental Neuroscience 31(4):300–8. 10.1159/000216541. 19546567

[R56] Okbay Aysu, Benjamin Daniel, and Visscher Peter. 2018. “Documentation.” [Construction of Wisconsin Longitudinal Study Polygenic Scores.] University of Wisconsin— Madison. https://www.ssc.wisc.edu/wlsresearch/documentation/GWAS/Lee_et_al_(2018)_PGS_WLS.pdf.

[R57] Oliynyk Roman Teo. 2019. “Age-Related Late-Onset Disease Heritability Patterns and Implications for Genome-Wide Association Studies.” PeerJ 7:e7168. 10.7717/peerj.7168. 31231601 PMC6573810

[R58] Pearson Thomas A., and Manolio Teri A.. 2008. “How to Interpret a Genome-Wide Association Study.” JAMA 299(11):1335–44. 10.1001/jama.299.11.1335. 18349094

[R59] Pirastu Nicola, Cordioli Mattia, Nandakumar Priyanka, Mignogna Gianmarco, Abdellaoui Abdel, Hollis Ben, Kanai Masahiro, Rajagopal Veera Manikandan, Della Briotta Parolo Pietro, Baya Nikolas, Carey Caitlin, Karjalainen Juha, Als Thomas D., Van der Zee Matthijs D., Day Felix R., Ong Ken K., Finngen Study, Me Research Team, Consortium iPSYCH, Morisaki Takayuki, de Geus Eco, Bellocco Rino, Okada Yukinori, Børglum Anders D., Joshi Peter, Auton Adam, Hings David, Neale Benjamin M., Walters Raymond K., Nivard Michel G., Perry John R. B., and Ganna Andrea. 2020. “Genetic Analyses Identify Widespread Sex-Differential Participation Bias.” bioRxiv. Preprint, submitted March 23. https://www.biorxiv.org/content/10.1101/2020.03.22.001453v1. 10.1038/s41588-021-00846-7 PMC7611642 33888908

[R60] Polderman Tinca, Benyamin Beben, De Leeuw Christiaan A., Sullivan Patrick F., Van Bochoven Arjen, Visscher Peter M., and Posthuma Danielle. 2015. “Meta-Analysis of the Heritability of Human Traits Based on Fifty Years of Twin Studies.” Nature Genetics 47(7):702–9. 10.1038/ng.3285. 25985137

[R61] Purcell Shaun. 2002. “Variance Components Models for Gene–Environment Interaction in Twin Analysis.” Twin Research and Human Genetics 5(6):554–71. 10.1375/136905202762342026. 12573187

[R62] Rosenquist James Niels, Lehrer Steven F., O’Malley A. James, Zaslavsky Alan M., Smoller Jordan W., and Christakis Nicholas A.. 2015. “Cohort of Birth Modifies the Association between FTO Genotype and BMI.” Proceedings of the National Academy of Sciences 112(2):354–59. 10.1073/pnas.1411893111. PMC4299180 25548176

[R63] Sugrue Leo P., and Desikan Rahul S.. 2019. “What Are Polygenic Scores and Why Are They Important?” JAMA 321(18):1820–21. 10.1001/jama.2019.3893. 30958510

[R64] Taylor Amy E., Jones Hannah J., Sallis Hannah, Euesden Jack, Stergiakouli Evie, Davies Neil M., Zammit Stanley, Lawlor Debbie A., Munafò Marcus R., Smith George Davey, and Tilling Kate. 2018. “Exploring the Association of Genetic Factors with Participation in the Avon Longitudinal Study of Parents and Children.” International Journal of Epidemiology 47(4):1207–16. 10.1093/ije/dyy060. 29800128 PMC6124613

[R65] Trejo Sam, Belsky Daniel, Boardman Jason, Freese Jeremy, Harris Kathleen, Herd Pam, Sicinski Kamil, and Domingue Benjamin. 2018. “Schools as Moderators of Genetic Associations with Life Course Attainments: Evidence from the WLS and Add Health.” Sociological Science 5:513–40. 10.15195/v5.a22. 30613760 PMC6314676

[R66] Tropf Felix C., Lee S. Hong, Verweij Renske M., Stulp Gert, van der Most Peter J., de Vlaming Ronald, Bakshi Andrew, Briley Daniel A., Rahal Charles, Hellpap Robert, Iliadou Anastasia N., Esko Tõnu, Metspalu Andres, Medland Sarah E., Martin Nicholas G., Barban Nicola, Snieder Harold, Robinson Matthew R., and Mills Melinda C.. 2017. “Hidden Heritability Due to Heterogeneity across Seven Populations.” Nature Human Behaviour 1:757–65. 10.1038/s41562-017-0195-1. PMC5642946 29051922

[R67] Troth Ashley, Puzey Joshua R., Kim Rebecca S., Willis John H., and Kelly John K.. 2018. “Selective Trade-Offs Maintain Alleles Underpinning Complex Trait Variation in Plants.” Science 361(6401):475–78. 30072534 10.1126/science.aat5760

[R68] Tucker-Drob Elliot M. 2017. “Measurement Error Correction of Genome-Wide Polygenic Scores in Prediction Samples.” bioRxiv. Preprint, submitted July 19. https://www.biorxiv.org/content/10.1101/165472v1.

[R69] van der Wal Willem M., and Geskus Ronald B.. 2011. “IPW: An R Package for Inverse Probability Weighting.” Journal of Statistical Software 43(13):1–23. 10.18637/jss.v043.i13.

[R70] Visscher Peter M., Wray Naomi R., Zhang Qian, Sklar Pamela, McCarthy Mark I., Brown Matthew A., and Yang Jian. 2017. “10 Years of GWAS Discovery: Biology, Function, and Translation.” American Journal of Human Genetics 101(1):5–22. 10.1016/j.ajhg.2017.06.005. 28686856 PMC5501872

[R71] Wang Huanwei, Zhang Futao, Zeng Jian, Wu Yang, Kemper Kathryn E., Xue Angli, Zhang Min, Powell Joseph E., Goddard Michael E., Wray Naomi R., Visscher Peter M., McRae Allan F., and Yang Jian. 2019. “Genotype-by-Environment Interactions Inferred from Genetic Effects on Phenotypic Variability in the UK Biobank.” Science Advances 5(8):eaaw3538. 10.1126/sciadv.aaw3538. 31453325 PMC6693916

[R72] Ware Erin B., Schmitz Lauren L., Faul Jessica D., Gard Arianna, Mitchell Colter, Smith Jennifer A., Zhao Wei, Weir David, and Kardia Sharon L. R.. 2017. “Heterogeneity in Polygenic Scores for Common Human Traits.” bioRxiv. Preprint, submitted February 5. https://www.biorxiv.org/content/10.1101/106062v1.

[R73] Widaman Keith F., Helm Jonathan L., Castro-Schilo Laura, Pluess Michael, Stallings Michael C., and Belsky Jay. 2012. “Distinguishing Ordinal and Disordinal Interactions.” Psychological Methods 17(4):615–22. 10.1037/a0030003. 22984788 PMC3553243

[R74] Yang J, Lee SH, Goddard ME, and Visscher PM. 2011. “GCTA: A Tool for Genome-Wide Complex Trait Analysis.” American Journal of Human Genetics 88(1):76–82. 10.1016/j.ajhg.2010.11.011. 21167468 PMC3014363

[R75] Yang Jian, Loos Ruth J. F., Powell Joseph E., Medland Sarah E., Speliotes Elizabeth K., Chasman Daniel I., Rose Lynda M., Thorleifsson Gudmar, Steinthorsdottir Valgerdur, Magi Reedik, Waite Lindsay, Smith Albert Vernon, Yerges-Armstrong Laura M., Monda Keri L., Hadley David, Mahajan Anubha, Li Guo, Kapur Karen, Vitart Veronique, Huffman Jennifer E., Wang Sophie R., Palmer Cameron, Esko Tonu, Fischer Krista, Jing Hua Zhao Ayse Demirkan, Isaacs Aaron, Feitosa Mary F., Jian’an Luan Nancy L. Heard-Costa, White Charles, Jackson Anne U., Preuss Michael, Ziegler Andreas, Eriksson Joel, Kutalik Zoltan, Frau Francesca, Nolte Ilja M., Van Vliet-Ostaptchouk Jana V., Hottenga Jouke-Jan, Jacobs Kevin B., Verweij Niek, Goel Anuj, Medina-Gomez Carolina, Estrada Karol, Bragg-Gresham Jennifer Lynn, Sanna Serena, Sidore Carlo, Tyrer Jonathan, Teumer Alexander, Prokopenko Inga, Mangino Massimo, Lindgren Cecilia M., Assimes Themistocles L., Shuldiner Alan R., Hui Jennie, Beilby John P., McArdle Wendy L., Hall Per, Haritunians Talin, Zgaga Lina, Kolcic Ivana, Polasek Ozren, Zemunik Tatijana, Oostra Ben A., Gronberg Henrik, Schreiber Stefan, Peters Annette, Hicks Andrew A., Stephens Jonathan, Foad Nicola S., Laitinen Jaana, Pouta Anneli, Kaakinen Marika, Willemsen Gonneke, Vink Jacqueline M., Wild Sarah H., Navis Gerjan, Asselbergs Folkert W., Homuth Georg, John Ulrich, Iribarren Carlos, Harris Tamara, Launer Lenore, Gudnason Vilmundur, O’Connell Jeffrey R., Boerwinkle Eric, Cadby Gemma, Palmer Lyle J., James Alan L., Musk Arthur W., Ingelsson Erik, Psaty Bruce M., Beckmann Jacques S., Waeber Gerard, Vollenweider Peter, Hayward Caroline, Wright Alan F., Rudan Igor, Groop Leif C., Metspalu Andres, Khaw Kay Tee, van Duijn Cornelia M., Borecki Ingrid B., Province Michael A., Wareham Nicholas J., Tardif Jean-Claude, Huikuri Heikki V., Cupples L. Adrienne, Atwood Larry D., Fox Caroline S., Boehnke Michael, Collins Francis S., Mohlke Karen L., Erdmann Jeanette, Schunkert Heribert, Hengstenberg Christian, Stark Klaus, Lorentzon Mattias, Ohlsson Claes, Cusi Daniele, Staessen Jan A., Van der Klauw Melanie M., Pramstaller Peter P., Kathiresan Sekar, Jolley Jennifer D., Ripatti Samuli, Jarvelin Marjo-Riitta, de Geus Eco J. C., Boomsma Dorret I., Penninx Brenda, Wilson James F., Campbell Harry, Chanock Stephen J., van der Harst Pim, Hamsten Anders, Watkins Hugh, Hofman Albert, Witteman Jacqueline C., Zillikens M. Carola, Uitterlinden Andre G., Rivadeneira Fernando, Zillikens M. Carola, Kiemeney Lambertus A., Vermeulen Sita H., Abecasis Goncalo R., Schlessinger David, Schipf Sabine, Stumvoll Michael, Tonjes Anke, Spector Tim D., North Kari E., Lettre Guillaume, McCarthy Mark I., Berndt Sonja I., Heath Andrew C., Madden Pamela A. F., Nyholt Dale R., Montgomery Grant W., Martin Nicholas G., McKnight Barbara, Strachan David P., Hill William G., Snieder Harold, Ridker Paul M., Thorsteinsdottir Unnur, Stefansson Kari, Frayling Timothy M., Hirschhorn Joel N., Goddard Michael E., and Visscher Peter M.. 2012. “FTO Genotype Is Associated with Phenotypic Variability of Body Mass Index.” Nature 490(7419):267–72. 10.1038/nature11401. 22982992 PMC3564953

[R76] Young Alexander I., Wauthier Fabian L., and Donnelly Peter. 2018. “Identifying Loci Affecting Trait Variability and Detecting Interactions in Genome-Wide Association Studies.” Nature Genetics 50(11):1608–14. 10.1038/s41588-018-0225-6. 30323177

[R77] Zajacova Anna, and Burgard Sarah A.. 2013. “Healthier, Wealthier, and Wiser: A Demonstration of Compositional Changes in Aging Cohorts Due to Selective Mortality.” Population Research and Policy Review 32(3):311–24. 10.1007/s11113-013-9273-x. 25075152 PMC4112120

